# Limited Spread of Penicillin-Nonsusceptible Pneumococci, Skåne County, Sweden

**DOI:** 10.3201/eid1006.030488

**Published:** 2004-06

**Authors:** Eva Melander, Hans-Bertil Hansson, Sigvard Mölstad, Kristina Persson, Håkan Ringberg

**Affiliations:** * Department of Clinical Microbiology, Lund University Hospital, Lund, Sweden;; † Regional Center for Communicable Disease Control, Skåne County, Sweden;; ‡ Unit of Research and Development in Primary Health Care, Jönköping County, Sweden

**Keywords:** Streptococcus pneumonia, drug resistance, microbial, antibiotics, children, community, drug utilization, intervention studies

## Abstract

In response to increasing frequencies of penicillin-nonsusceptible pneumococci (PNSP), for which the MIC of penicillin was >0.12 mg/L, in Skåne County, southern Sweden, national recommendations were initiated in 1995 to limit the spread of pneumococci with high MICs (>0.5 mg/L) of penicillin (PRP), especially among children of preschool age. Traditional communicable disease control measures were combined with actions against inappropriate antimicrobial drug use. During the first 6 years that these recommendations were applied in Skåne County, the average frequency of penicillin-resistant pneumococci has been stable at ≈2.6%, as has the average PNSP frequency (7.4%). However, PNSP have been unevenly distributed in the county, with the highest frequencies in the southwest. Simultaneously, the rate of antimicrobial drug use for children <6 years of age was reduced by 20%. Thus the spread of PNSP between and within the municipalities in the county has been limited.

Increasing frequencies of penicillin-nonsusceptible pneumococci (PSNP) (MIC of penicillin >0.12 mg/L) became a worldwide problem in the 1980s (*1**,**2*). The rapid increase in frequency is likely caused by intercontinental spread of a few PNSP clones for which MICs of penicillin are high (*3**–**6*). For a long period, very low PNSP frequencies were noted in Sweden (2%–3%) (*7*). However, in the early 1990s, the incidence of PNSP increased from 8% to 10% in Skåne, the southernmost county, while the rest of the country reported unchanged low frequencies (*8**–**10*). When considered from the perspective of international experience, in which the rate of PNSP rapidly increased when this level of resistance was reached (*11*), the increasing frequencies of PNSP in Skåne County led to the formation of an expert committee, appointed by the National Board of Health and Welfare. This committee proposed a national strategy in 1995, based on reducing unnecessary use of antimicrobial agents and applying infection control measures to limit the more immediate spread of PNSP for which MICs of penicillin were high (>0.5 mg/L, PRP), especially in preschool children ages 1 to 6 years. In 1996, infection and carriage with PRP became notifiable by the Swedish Communicable Disease Act. The reasons to choose MIC >0.5 mg/L as a limit for intervention were that epidemiologic data on pneumococci suggested that an increased prevalence of strains for which the MIC of penicillin was >1.0 mg/L most often was caused by spread of a few already resistant clones, so that those strains could lead to treatment failures, and that those strains were relatively rare in Sweden in 1994 (*12*). Since the Etest can be hard to interpret, MIC >0.5 mg/L was chosen so strains for which the MIC of penicillin was 1.0 mg/L would not be missed. The decision of whether to follow the recommendations of the expert committee is made by each county department for communicable disease control. The recommendations were strictly applied in Skåne County (South Swedish Pneumococcal Intervention Project, SSPIP), while some Swedish counties either did not follow the recommendations at all or only applied parts of the recommendations. For a long period, Skåne County had the highest use of antimicrobial agents in Sweden, especially of macrolides and broad-spectrum antibiotics (*13*), and in the SSPIP traditional communicable disease control measures are combined with actions aimed at reducing the use of antimicrobial drugs. Experiences from the first 2 years of the SSPIP from parts of Skåne County have previously been reported (*14**–**17*). For example, data on individual risk factors for carriage of PNSP have been evaluated. We discuss the overall results of the SSPIP and evaluate the effects of the recommendations in Skåne County during the first 6 years they were implemented.

## Materials and Methods

### Study Area

On January 1, 2000, the population of Skåne County was 1,129,424 inhabitants; 93,051 were <6 years of age. The county is divided into 33 municipalities. The largest municipality, Malmö, had 259,579 inhabitants and of those, 20,325 were <6 years of age. The smallest municipality had 6,745 inhabitants; 581 were <6 years. During the study period, ≈76% of the children 1 to 6 years of age were enrolled in day care.

### SSPIP

The principles of the SSPIP have been described earlier (*16*). In brief, all persons in Skåne County with a culture that yields penicillin-nonsusceptible pneumococci with an MIC > 0.5mg/L for penicillin (PRP), regardless of resistance to any other antibiotics, have since 1995 been reported to the Regional Center of Communicable Disease Control (CCDC). Whenever a person with a clinical infection caused by PRP (index case-patient) is identified, nasopharyngeal cultures are obtained from family members and other close contacts to identify asymptomatic PRP carriers (contact case-patients). All carriers are followed weekly with nasopharyngeal cultures at the local primary health care center until two consecutive cultures that yield no growth of PRP (PRP-negative) have been obtained. If the index case is in a child attending any form of group day care, nasopharyngeal cultures are obtained from the other children and staff members at that day care group. Preschool children are restricted from attending day care until they are PRP-negative. In selected cases, eradication therapy with antimicrobial drugs is considered after 2 to 3 months of carriage, or earlier when strong social reasons exist (*18*).

### Microbiologic Methods

The pneumococcal strains considered in this study were recovered from cultures analyzed at the Departments of Clinical Microbiology in Lund, Malmö, Helsingborg, and Kristianstad from July 1, 1995, through June 30, 2001. These four laboratories served the entire population of Skåne County during the study period. The specimen were cultured on blood agar plates, and the isolates were identified as *Streptococcus pneumoniae* on the basis of colony morphology and susceptibility to optochin (*19*). The strains were screened for penicillin-resistance by using the disk-diffusion method, according to the Swedish Reference Group for Antibiotics (SRGA). The strains were inoculated onto Iso Sensitest agar (Oxoid Ltd, Basingstoke, UK), supplemented according to the recommendations, and the antibiotic disks (Oxoid Ltd) were applied. Inhibition zones were read to the nearest millimeter and interpreted according to SRGA guidelines (*20*). For pneumococci with an oxacillin 1 mg inhibition zone <20 mm, the MIC of penicillin was determined by the Etest (AB Biodisk, Solna, Sweden) (*21*). The susceptibility to other antimicrobial agents (erythromycin, tetracycline, trimethoprim-sulfamethoxazole, and clindamycin) was determined by using the disk diffusion method (*10*). Strains from each patient were registered only once per season, even if multiple cultures were positive for pneumococci. Whether the strains were recovered at a visit to the doctor or by contact tracing or screening was noted. Serotyping to the group level was performed by the quellung reaction, by using antisera from the Statens Seruminstitut, Copenhagen, Denmark (*19*).

### Antimicrobial Drug Use

Data were collected regarding antimicrobial drug prescriptions for outpatient care, served at Swedish pharmacies from July 1, 1995, through June 30, 2001, that were issued for children ages <6 years who lived in Skåne County; these data were obtained from the Corporation of Swedish Pharmacies, which owns all Swedish pharmacies and collects and compiles information on all drugs sold in the country (*13*). The municipality of the patient was registered. The prescribed antimicrobial agents were given as prescriptions per 1,000 inhabitants ages <6 years per season, and all prescriptions of phenoxymethylpenicillin (PcV), ampicillin/amoxicillin (including amoxicillin+clavulanic acid), cephalosporins, trimethoprim-sulfamethoxazole, macrolides, clindamycin, and other antimicrobial drugs (grouped together) were registered.

## Results

**Figure 1:**
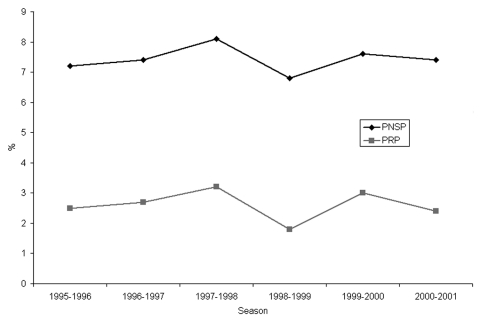
Frequency of penicillin-nonsusceptible pneumococci with MIC for penicillin >0.12 mg/L (PNSP) and >0.5 mg/L (PRP) per season in Skåne County, Sweden, expressed in percentage of all pneumococci from clinical nasopharyngeal cultures.

During the project, the frequency of PNSP carriers from clinical nasopharyngeal cultures has been evaluated. Cultures taken at contact tracing were excluded. From July 1, 1995, until June 30, 2001, the average frequency of PNSP carriers has been stable, approximately 7.4% (2,750 PNSP/34,745 pneumococci) (Figure 1). The highest frequencies (8.1%) were seen in 1997 to 1998. During the 6 years of the recommendations, the levels of PNSP have been unevenly distributed over the county; higher and stable levels were found in the city of Malmö in the southwestern part of the county, (11.3%, 976 PNSP/8,651 pneumococci) and lower, but stable, levels were found in the northeastern part of the county (4.3%, 395 PNSP/9,109 pneumococci). The frequency of PNSP among invasive pneumococcal strains (blood and cerebrospinal fluid cultures) in Skåne County has been low, ≈2.5 % (22 PNSP/858 pneumococci). As with the PNSP in nasopharyngeal cultures, the highest frequencies of invasive PNSP were seen in 1997 to 1998.

The average frequency of index case-patients with PRP (that is, the number of clinical case-patients with a culture with growth of PRP, divided by the number of pneumococci found in nasopharyngeal cultures) has been rather stable since the start of the project, ≈2.6% (905 PRP/34,745 pneumococci) (Figure 1). In the southwestern part of the county, in the cities of Malmö and Lund and surrounding municipalities, where most cases were found, PRP have been constantly present. However, in several of the smaller municipalities, PRP were prevalent during one or two seasons but then disappeared. In two municipalities, both situated in the northeast, no cases of PRP infection have been found. The proportion of cases with PRP with high MICs (>2 mg/L) of penicillin has not increased during the 6 years (Table). Carriers of PRP with MIC >4.0 mg/L have all been adopted children or immigrants from Eastern Europe or from countries outside Europe.

**Table:** Distribution of MICs of penicillin for all registered PRP cases in Skåne County per season^a^

Season	0.5 mg/L n (%)	1.0 mg/L n (%)	>2.0 mg/L n (%)
1995–1996	217 (33)	290 (44)	151 (23)
1996–1997	132 (36)	140 (37)	102 (27)
1997–1998	189 (42)	214 (47)	52 (11)
1998–1999	124 (46)	140 (52)	4 (2)
1999–2000	183 (59)	111 (35)	18 (6)
2000–2001	124 (62)	75 (38)	0

^a^PRP, pneumococci with MIC >0.5 mg/L).

During the 6-year period, 2,269 PRP carriers (1,865 persons) have been registered at the CCDC in Skåne County. Of the 2,269 PRP carriers, 40% were index case-patients, and 60% were contacts. Few contact cases have been found in persons at the extremes of ages (£1 year and >65 years). The number of contact cases per index patients in children <6 years of age has, on an average, been 1.6 (1.1 to 2.2 per season) and has not increased. The index patients have most often been found through positive nasopharyngeal cultures, and only a minority (n = 13, 1.4%) have been found through positive blood or cerebrospinal fluid cultures. More men carried PRP overall, but in the group ages 18 to 64 years, PRP carriage was more common among women. A clear seasonal variation in the incidence of PRP was found; considerably more cases occurred during the winter months (October–March) than during summer. The median duration of PRP carriage was 21 days (range 2–368 days); 1,704 (75%) of the PRP patients were children 1 to 6 years of age. Seventy-one percent of these children attended day care centers; 6% attended family day care, and 23% were cared for at home. The other contacts have primarily been siblings, parents, or grandparents of young children.

Two hundred and twenty-seven children with a clinical culture showing growth of PRP, led to screening of children and staff at 227 of the county’s 1,250 day care centers. The number of contact cases per index case-patient among all children attending these day care centers has, on an average, been 3.46 (2.6–4.2 per season) and has not increased, and the number of contact cases in each day care center has varied from 0 to 25 (median 2). In 24% of the day care center interventions, no contact cases were found. In 45 of the 227 day care centers, more than one PRP serotype was discovered, and 65 day care centers have been investigated twice or more (median 2, range 2–6). Twenty-two of these day care centers had two outbreaks with the same serotype within 6 months. Most of the day care interventions took place in the southwestern part of the county. Only 20 of all screened staff at day care centers were PRP-positive.

Serotyping to the group level was performed for 2,131 (94%) of the 2,269 PRP strains. Twenty-five serotypes of PRP were found. Six serotypes comprised 93% of the strains (serotypes 9, 19, 6, 23, 15, 14). The most prevalent strain, serotype 9, represented an average of 48% of the PRP strains (37%–66% per season). This strain has spread over the county from municipality to municipality in a clear pattern, whereas the other common serotypes have spread more randomly.

**Figure 2:**
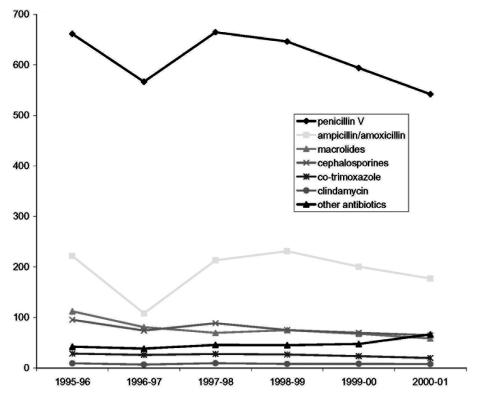
Use of antimicrobial agents in outpatient care among children ages <6 years in Skåne County, Sweden, expressed in prescriptions per 1,000 inhabitants per season.

The use of antimicrobial agents in outpatient care in Skåne County decreased from the first to the last season in all age groups, but especially in children aged <6 years (Figure 2). The main reduction among children ages <6 years was caused by a decrease in number of prescriptions for phenoxymethylpenicillin, and the use of macrolides was reduced by 50%. Even though the decreased use of antimicrobial drugs was seen in all municipalities, the municipalities with the highest utilization in 1995–1996 still had the highest use in 2000–2001. Those municipalities were all located in the southwestern part of the county.

## Discussion

In Skåne County, where PNSP (pneumococci with an MIC for penicillin >0,12 mg/L) have been prevalent for >10 years, the frequency of PNSP in clinical nasopharyngeal cultures (cultures taken at contact tracing excluded) remained at the same level in 2000 to 2001 as in 1995 to 1996, on average, 7.4%. In the city of Malmö, where the spread of PNSP appears to have started, the average level of PNSP has been higher (11.3%) during the project period. In contrast, the average level of PNSP remained low (4.3%) during the study period in the northern and eastern parts of the county. Furthermore, the number of PRP contact cases per index case among children attending day care centers did not increase during the 6-year period. These data indicate that the rapid spread of PNSP that started in the southwestern part of the county in the beginning of the 1990s (*8**,**9*) has been limited. These results are unique in comparison with experience in other countries, where PNSP rates have rapidly increased after reaching an 8% PNSP level (*11*). The proportion of contacts with PRP with high MICs of the PNSP in Skåne County has not increased during the 6-year period, contrary to results from most other countries, which report increasing frequencies of PNSP with high MICs (*22**,**23*).

One of the project’s aims was to decrease unnecessary use of antimicrobial agents, especially of macrolides and trimethoprim-sulfamethoxazole, which in studies have been shown to promote spread of PNSP (*17**,**24**–**27*). During the study period, the use of antimicrobial drugs decreased in all municipalities in the county, and the decrease was most prominent in children ages <6 years. In this age group, the use of macrolides was cut in half. The use of long-acting macrolides has been extremely low and has comprised <3% of the total use of macrolides among children. Even though the use of antimicrobial agents decreased in all municipalities of Skåne County, those with high usage in 1995 to 1996 also had high usage in 2000 to 2001, and those with low usage in 1995 to 1996 had an even lower usage in 2000 to 2001. Mirroring the use of antimicrobial agents, the occurrence of PNSP has been unevenly distributed in the county, with constant high rates in the southwestern part and low levels in the northern and eastern parts. In cities in the northeastern parts and in the smaller municipalities, with lower antimicrobial agent use, single outbreaks have occurred without any more cases being reported later. A possible explanation for this might be that municipalities in which antibiotics are frequently used have a higher risk of spreading PNSP in the community, as indicated by previously published data from the project as well as by other studies (*17**,**24**,**28**,**29*).

Many PRP serotypes were found in the county during the six seasons. However, one serotype, serotype 9, has dominated during all six seasons and has spread in a clear pattern from municipality to municipality throughout the county (*15*), while other strains have spread more randomly in time and geographically. Why this strain has been persistently present for such a long time is unclear.

We chose to present the figures for the frequency of PNSP in clinical cultures over time, excluding cultures taken at contact tracing, since the contacts were not found at random, but rather represented a selected population around an index patient. The exclusion of cultures taken at contact tracing ought to give more fair figures when comparing data from the study period with the "resistance situation" before the project started or with data from other studies. The possibility of excluding all cultures taken at contact tracing retrospectively was not 100% guaranteed, but cultures taken at day care center screenings were easily found and excluded. The rest of the cultures obtained because of contact tracing constituted only a small proportion of cultures, and thus they should not be a source of error.

Although the indication for nasopharyngeal sampling was not changed during the study period, the number of obtained samples has decreased since the start of the project. However, the number of nasopharyngeal cultures increased in 1995 to 1996 compared to 1993 to 1994. One possible explanation for this might be that the health authorities encouraged doctors to take nasopharyngeal samples more frequently during the first years of the project.

**Figure 3:**
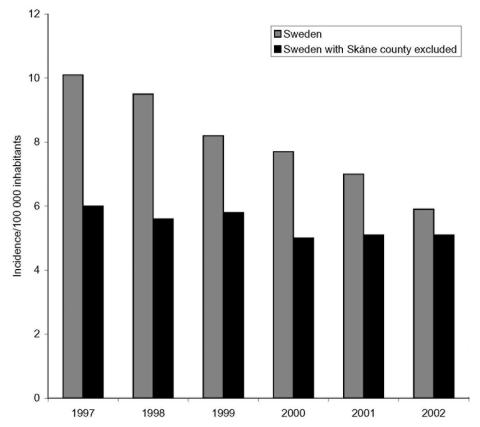
National incidence of penicillin-resistant pneumococci with and without Skåne County included.

Exactly comparable data on PNSP frequencies from other Swedish counties or comparable survey data (nasopharyngeal cultures) from other countries are hard to find. However, since 1996, Swedish law has mandated the reporting of all PRP strains to the Swedish Institute for Infectious Disease Control. Data from this national register contains information on reported PRP from all over Sweden from 1997 to 2002. Of all reported PRP, the PRP from Skåne County comprised 40% in 1997, but only 10% in 2002 (Figure 3). Furthermore, the recommendations are strictly applied in Skåne County, while some Swedish counties either do not follow the recommendations at all or only apply parts of the recommendations. In some counties, where the recommendations were not applied or not as strictly applied as in Skåne County, the frequencies of PRP have increased from 2000 to 2002 (*30**,**31*). Moreover, according to data from the annual Resistance Surveillance and Quality Control Programme, the frequency of PNSP in clinical nasopharyngeal cultures in Sweden has increased from 3.8% in 1994 to 6.2% in 2002 (*30**,**32*). In addition, the frequency of PNSP among invasive pneumococcal strains in Sweden has, on average, increased from 1.4% in 1999 to 2.4% in 2002 (*30**,**33*), but the frequency of invasive PNSP during the same period in Skåne County has been approximately 2.5% and stable. The numbers of invasive PNSP isolates in Skåne County are very low, and therefore drawing any reliable conclusions from these data is difficult. However, the frequency of PNSP has not increased, as it has in most other countries, and the trends of invasive PNSP seem to follow the trends for PNSP from nasopharyngeal cultures. Compared to the other Nordic countries, who still report low frequencies of invasive PNSP, Sweden has had the lowest frequencies of invasive PNSP between 1999 and 2002 (*33*).

Although the frequencies of PNSP in Skåne County were not reduced, the spread of PNSP seems to have been limited. Whether this is a result of the actions of the SSPIP among children or of the decreased use of antibiotics in the county, or both, is impossible to tell since these actions were started simultaneously. In other countries, actions against unnecessary use of antimicrobial drugs has been the only measure of combating the spread of PNSP, in most cases with little success, probably in part because the actions have been initiated in a much later phase, when frequencies of PNSP were higher (*34**–**37*). Still, further efforts are necessary to reduce the prescribing of antimicrobial agents in the southwestern areas, since the use of antimicrobial agents is still high there and poses the greatest risk factor for PNSP carriage.
